# Bridging the gap between science, policy, stakeholders: Delineating a virtuous framework for agriculture under climate change scenarios

**DOI:** 10.1016/j.isci.2025.113326

**Published:** 2025-08-08

**Authors:** Aurora Ghirardelli, Paolo Tarolli

**Affiliations:** 1 Department of Land, Environment, Agriculture and Forestry, University of Padova, Padua, Italy

**Keywords:** Environmental science, Environmental policy, Agricultural science, Agricultural policy, Relation between agriculture and environment

## Abstract

Addressing the challenge of feeding a growing global population while mitigating the damages of weather extremes and adapting to climate change requires coordinated efforts across science, policy, and agriculture. Drawing inspiration from recent European Union agricultural and environmental policy reforms, we examine the barriers between scientific advancements, farm-scale applications, and the implementation of agricultural policies. We propose a generalized framework to bridge communication gaps among scientists, policymakers, and farmers. By fostering participatory research, empowering stakeholders, and enhancing education, while still acknowledging the importance of economic efficiency, the framework aims to align environmental goals with farmers’ socio-economic realities, and strengthen their active role in environmental protection. It highlights the need to overcome technological and economic barriers, enhance scientific understanding of regional environmental issues, and strengthen collaboration within the scientific community. This approach aims to ensure sustainable agricultural practices, building resilience to climate change while balancing productivity and environmental conservation.

## Graphical abstract

**Figure:**
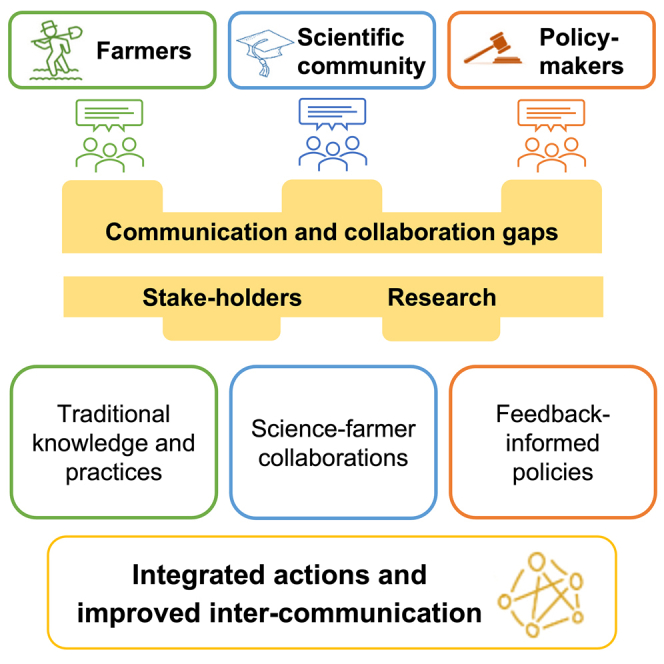

## Introduction

Agricultural production is essential for sustaining the livelihoods of a global population projected to reach 10 billion by 2050. Meeting the dietary demands of this demographic expansion will require a 50% increase in food production compared to 2013 levels.^1^ Consequently, food security is a cornerstone of the United Nations’ Sustainable Development Goals (SDGs), which aim to guide countries toward sustainable socio-economic and environmental progress.^2^ However, despite advancements in farming efficiency, yield growth has slowed due to anthropogenic factors, such as land degradation, inefficient water management, increasing pest resistance to agrochemicals, and biodiversity loss. In addition to these challenges, climate change poses the greatest threat to agriculture, exacerbating extreme weather events, such as severe droughts, intense rainfall, cyclones, and accelerating sea-level rise.^1^ Therefore, agricultural productivity is hampered by abiotic stresses, environmental conditions that negatively impact plant growth, survival, and reproduction.^3^^,^^4^ Key abiotic stressors include heat, drought, flooding, high light intensity, heavy metal exposure, and soil salinity. The combined pressures of population growth, climate-induced disruptions, and other human-driven stressors are intensifying global food shortages and widening the gap between production and demand.^5^^,^^6^^,^^7^

Rising global temperatures have significantly intensified the hydrological cycle, increasing the frequency and severity of extreme precipitation events.^8^ Both climate models and historical data indicate that these changes are disrupting precipitation patterns worldwide. In many regions, seasonal precipitation has shifted toward severe storms concentrated over short periods, interspersed with extended dry spells.^9^ This imbalance triggers natural hazards, such as floods, landslides, and soil erosion, which inflict casualties and economic losses, particularly in agriculturally intensive areas. On the other hand, prolonged periods without precipitation, combined with high temperatures and low soil water content, have led to increased drought frequency and severity.^10^ A concerning development is the rise of flash droughts, which occur suddenly and leave little time for mitigation or prevention.^11^ These abrupt events are expected to become more frequent, intensifying the challenges for agriculture and society. Overall, the intensification of extreme weather events is having more severe consequences than in the past due to higher exposure and vulnerability. Anthropogenic changes to landscapes, such as urbanization and altered river systems, are contributing to the increased complexity of geo-hydrological risks.^12^ Mountainous and lowland riverine systems are especially vulnerable to geo-hydrological disruptions caused by intense rainfall, while coastal regions are particularly exposed to salinization caused by seawater intrusion and coastal erosion due to coastal storms.^12^^,^^13^

**Figure 1:**
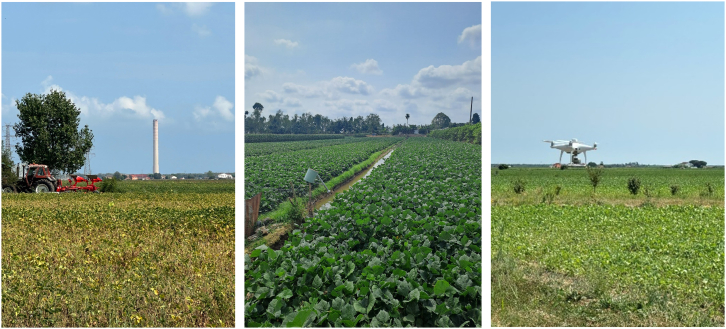
Three approaches to agricultural management Mechanized and high-input agriculture, traditional practices, and science-driven innovation tools. Photos taken in Porto Tolle (Italy) and Cần Thơ (Vietnam) by: Vincenzo Baldan, Aurora Ghirardelli, and Paolo Tarolli.

Addressing the challenge of feeding a growing global population while mitigating and adapting to climate change requires coordinated efforts across science, policy, and practice, and might lead to different approaches to agricultural management (Figure 1). Developing effective landscape management strategies and implementing consistent risk mitigation measures are crucial steps to enhance resilience and secure the future of agricultural systems in a rapidly changing world.^12^ The scientific community must focus on understanding the future climate trends and processes and proposing and perfecting innovative strategies for sustainable agriculture under changing these scenarios, such as improving crop resilience to extreme weather events, enhancing water-use efficiency, and adopting nature-based solutions (NBSs). Stakeholders, particularly farmers and agribusinesses, navigating climate-induced risks while trying to maintain profitable activities, must develop strategies for sustainable water and soil management, implement precision agriculture technologies, and adopt adaptive practices to mitigate yield losses. Policymakers, meanwhile, face the complex challenge of designing effective climate policies that promote resilience and sustainability while addressing the immediate needs of agricultural communities. These policies must provide financial support, incentivize sustainable practices, and ensure equitable resource distribution, particularly in vulnerable regions.^14^ However, a significant barrier to progress lies in the communication gaps between these three groups. Scientists often struggle to translate research findings into actionable insights for farmers and decision-makers, and they tend to focus only on the ecological protection aspect, while not considering socio-economic sustainability. Farmers and other local stakeholders may lack access to the latest innovations or the means to implement them effectively.^15^^,^^16^ Traditional and cultural practices might present inherent resilience to extreme climate events^17^; however, they are frequently overlooked by the scientific community or undermined by decades of historical, one-size-fits-all agricultural policies.^18^

Many proposed frameworks emphasize participatory approaches that engage farmers in shaping tailored agricultural policies,^19^^,^^20^ and increasingly recognize the potential of agriculture to provide ecosystem services.^21^ However, despite evidence that farmers’ willingness to adopt adaptation measures is closely linked to their awareness of climate and environmental issue, particularly among those directly affected by climate change,^22^^,^^23^ such policies are often only partially effective.^20^^,^^24^ The purpose of this work is to explore the barriers between scientific advancements, practical farm-scale applications, and the implementation of environmental and agricultural policies, drawing insights from the recent European Union (EU) policy frameworks designed to reduce agriculture’s environmental footprint. We propose an integrated framework aimed at bridging communication gaps among scientists, policymakers, and farmers, to promote sustainable agriculture in the face of climate change, with particular emphasis on the potential farmers’ active contributions to large-scale environmental protection. Finally, we highlight the essential role of scientists in mediating between traditional practices and appropriate innovations, ensuring a balanced and context-sensitive approach.

## Mitigation and adaptation strategies: Technology and green solutions

### Innovations and applications from the scientific community

The threats posed by climate change and anthropogenic pressures on farming systems, particularly soil degradation and water scarcity, need proactive mitigation and adaptation strategies to secure a sustainable future for agriculture. In this sense, scientific advancements offer a multi-disciplinary toolbox of new and enhanced technologies for managing water and soil resources, enhancing ecosystem services, and improving resilience in farming systems. By combining science-driven innovations like remote sensing, artificial intelligence (AI) and NBS into integrated approaches, stakeholders can mitigate environmental impacts and initiate adaptation strategies. Remote and proximal sensing technologies, from satellite imagery to drones, robot- or vehicle-mounted sensors, have contributed to a change of paradigm in agricultural monitoring, providing more accurate data on soil moisture, crop health, pedological characterization, pest and weed infestations, allowing farmers to make data-driven, informed and targeted decisions. While historical coarse-resolution satellite data have advanced global uses like crop mapping and yield forecasting, recent technological progress has made fine-resolution data more accessible and practical.^25^^,^^26^ High-resolution imagery in the solar domain (multispectral reflectance and fluorescence) enables to optimize irrigation, reducing water wastage, and preventing plant stress due to abiotic stresses.^16^ Similarly, multi- and hyperspectral thermal infrared can detect water stress in crops, facilitating timely interventions to maintain yields.^26^ The availability of data at global (e.g., MODIS), regional (e.g., Sentinel-2), and canopy-leaf scales offers promising for integrating mechanistic models with machine learning to optimize real-time agricultural decision-making.^25^^,^^27^ Providing accessible information is crucial from global-to field-scale management, to address challenges linked to biodiversity loss, land degradation, and climate change and to adopt sustainable agricultural practices. Over the past decade, research has also progressed exploring the use of machine and deep learning on remote sensing datasets.^25^ AI applications are transforming farming practices, particularly during the growing, monitoring, and harvesting stages. AI-powered robots and devices have significantly advanced farming processes, enhancing precision and efficiency while minimizing human error and effort. Real-time data processed by AI enables better decision-making compared to traditional practices. Machine learning algorithms analyze data from sensors, weather forecasts, and historical trends to provide actionable insights. For instance, predictive models can help farmers determine the optimal time for sowing or harvesting based on weather conditions. Additionally, AI applications in robotic weeding and pest management reduce the need for agrochemicals, aligning agricultural practices with environmental sustainability goals. Key techniques such as fuzzy logic (FL) applied to decision and planning, artificial neural networks (ANN) used in complex and dynamic problem-solving, and genetic algorithms (GA) used as optimization tools, are widely utilized in agriculture.^28^^,^^29^ Currently, the use of standalone AI techniques dominates problem-solving in agriculture, however, the combination of multiple AI methods suggests potential enhancements in effectiveness. The use of robotics is particularly concentrated in the monitoring and harvesting phases, with fewer developments for cultivation tasks. Increasing focus on robotics for cultivation and diversifying the application of less commonly used AI techniques could further optimize agricultural operations.^29^ On the other hand, NBS are rising in popularity as cost-effective and sustainable alternatives to address climate challenges in agricultural and landscape management.^30^ In this sense, significant advancements have recently been made to enhance ecosystem functionality in landscapes impacted by agriculture and land degradation while supporting livelihoods and addressing social and cultural needs. These developments have expanded the range of NBS options, offering practical approaches to achieve conservation, climate resilience, and socioeconomic goals alongside maintaining productive agricultural systems.^31^ By employing or enhancing natural processes, land restoration and water management to enhance vegetation, they aim to improve water availability and quality, and boost agricultural productivity.^32^ In farming contexts, NBS can be particularly effective in improving soil health, retain soil moisture, mitigate carbon emissions (via soil management and forestry), protect downstream water quality, and promote biodiversity. Notable examples include wetland and lake restoration to absorb flood waters reducing flood risk, to enhance nutrient retention and cycling, and to serve as a natural barrier against soil salinization. Another common strategy that enhances biodiversity while providing economic benefits is agroforestry. For instance, the integration of hedgerows and buffer strips in farmland can reduce soil erosion, improve water retention, and create habitats for pollinators, contributing to both ecological and agricultural productivity.^30^^,^^31^ Another major set of NBS focuses on planting cover crops during fallow periods between primary crop cycles and maintaining continuous soil cover, minimizing soil disturbance, and diversifying plant species according to conservation agriculture principles. It supports biodiversity and fosters natural biological processes both above and below the soil surface. These processes enhance water and nutrient efficiency, contributing to improved and sustainable crop production.^33^ Integrated water and soil management strategies are another critical area of innovation strictly linked to NBS, and offer holistic solutions for sustaining agricultural systems. Resilient water management systems are vital for sustaining agricultural productivity and reducing adverse on-site and off-site environmental impacts. Strategies such as water harvesting and storage help mitigate the effects of prolonged droughts while preventing soil erosion, landslides, debris flow, and other degradation forms in mountain and hilly regions.^34^^,^^35^ During extreme rainfall events, effective water management practices, including ponds, terracing, and drainage systems are critical for controlling floods and soil erosion. A range of NBS like organic mulches, geotextiles, grass strips, soil bunds, can be applied to improve agricultural productivity, enhance water provision and capture sediment.^36^ High-steep slope terraced hillsides, among the most vulnerable agricultural systems, demand additional targeted strategies for effective erosion control and soil conservation over the long term. Managing the interaction between water and agricultural infrastructures, such as well-maintained dry-stone walls, is essential to ensure proper drainage and prevent structural failures caused by excessive water accumulation. Additionally, minimizing soil compaction by reducing heavy machinery use and employing techniques like soil ripping can improve water infiltration, promoting better water retention, and overall soil health.^37^ Another crucial strategy to optimize water use in dry conditions and reduce soil salinity in salinization-prone areas is the adoption of innovative irrigation techniques.^30^ Precision irrigation, supported by water-demand mapping, ensures efficient water application while minimizing over-irrigation risks. Advanced methods like micro-irrigation and subsurface drip irrigation deliver water directly to the root zone, reducing evaporation, preventing salt buildup around roots, and enhancing water efficiency. Additionally, automated irrigation systems powered by wireless sensor networks (WSN), the Internet of Things (IoT), and machine learning algorithms further enhance precision. These systems accurately monitor soil moisture using sensors, adapt irrigation schedules based on real-time crop and weather data, and prevent issues of over- or under-irrigation.^15^ All these advancements complement policy frameworks aiming to reduce agriculture’s carbon footprint while ensuring sustainable resource use, such as the EU Green Deal (EUGD) and Common Agricultural Policy (CAP).

### Environmental and agricultural strategies from policy-makers

In response to the escalating climate crisis, policymakers are increasingly focusing on frameworks that align agricultural practices with environmental and climate objectives. The EU has been at the forefront of this movement, with initiatives like the EGD and CAP efforts to transform the agricultural sector.^38^ The EUGD G represents a comprehensive strategy to achieve carbon neutrality by 2050, encompassing various sectors and strongly focusing on agriculture and agrifood systems. Key components relevant to farming include the “Farm to Fork” strategy, which aims to reduce pesticide use by 50% and increase organic farming practices, and the “Biodiversity Strategy,” which targets the restoration of degraded ecosystems.^39^^,^^40^ These initiatives emphasize sustainable resource use, reduced emissions, and enhanced resilience in agricultural landscapes.^41^ The EGD highlights the need for reforms in food production, agriculture, and broader land and resource management, based on the fact that agriculture is the second-largest contributor to greenhouse gas emissions in the EU, accounting for 11% of the total.^14^^,^^42^ In parallel, the CAP has evolved to integrate specific objectives for environmental protection and climate action. Its latest evolution (CAP 2023–2027) prioritizes reducing greenhouse gas emissions, preserving biodiversity, and improving soil and water quality. Through targeted subsidies and incentives, the CAP encourages farmers to adopt sustainable practices, such as crop rotation, agroforestry, and precision farming. Moreover “at least 25% of the budget for direct payments is allocated to eco-schemes, providing stronger incentives […] for farming practices and approaches such as organic farming, agro-ecology, carbon farming”.^43^ This is an example of how an intersection of scientific innovation and policy frameworks addresses both mitigation and adaptation challenges. By incorporating advanced technologies and green solutions into policy design, it is possible to amplify the impact of environmental goals while trying to maintain agricultural sustainability. However, obstacles remain in ensuring that agricultural policy objectives are effectively implemented and adapted to local contexts. The transition to greener agriculture involves multiple challenges, including sustaining crop yields, meeting nitrogen needs, managing land demand, adapting diets, and reducing food waste, requiring a holistic approach that integrates social, economic, cultural, technical, and environmental dimensions of the food system.^14^ Policies targeting climate protection and climate action, such as the EGD, struggle to guarantee socio-economic sustainability for farmers at all levels. Similarly, the scientific community encounters difficulties in transferring climate- and environmental-friendly innovations to stakeholders.

## Green solutions and socio-economic sustainability: Conflicts and communication gaps

### Challenges in socio-economic sustainability

**Figure 2:**
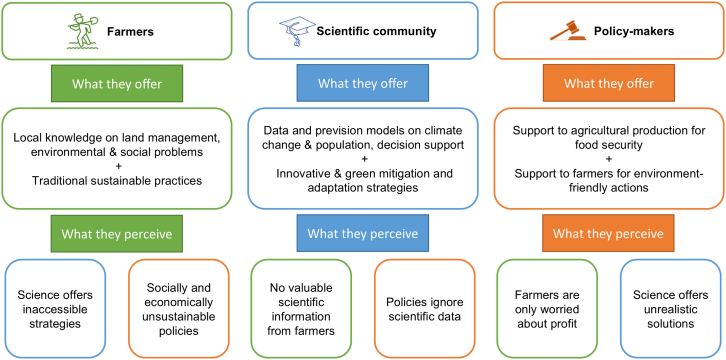
Representation of knowledge and communication divides between farmers, scientist, and policy-makers

Environmental and climate action policies are essential to address the pressing threats of climate change and environmental degradation. However, these policies often struggle to guarantee immediate socio-economic sustainability for farmers, particularly those in vulnerable regions or operating small-scale farms. While many environmental targets promise long-term benefits for agriculture, the short-term impacts can disproportionately burden those least equipped to adapt.^44^ Communication gaps between policymakers, scientists, and farmers are exacerbating these challenges and hindering the effective implementation of sustainable practices and equitable outcomes. Addressing these gaps is critical to achieving a balance between environmental goals and the socio-economic viability of agricultural communities (Figure 2). Environmental policies frequently aim at long-term benefits for farmers by promoting sustainable practices that improve soil health, enhance water management, and build resilience to climate change.^43^ For instance, crop rotation, a recommended practice in many environmental protection and climate action policies, can significantly enhance soil fertility, carbon sequestration and reduce dependency on chemical fertilizers.^45^^,^^46^ However, implementing such practices often demands short-term sacrifices that disproportionately affect small-scale farmers. In vulnerable areas where agriculture is already precarious due to climate variability, adopting new practices may lead to temporary reductions in yield or income, also due to lack of up-to-date scientific knowledge. For example, a small-scale farmer in a semi-arid or salinization-prone region might struggle to rotate crops effectively without access to irrigation infrastructure or subsidies for alternative crops, especially considering the different sensitivity levels to salt and water stress of each crop.^47^^,^^48^ Case studies from Sub-Saharan Africa show that farmers adopting crop rotation have faced initial income losses, as markets for non-traditional rotational crops were underdeveloped, leaving them with surplus produce and insufficient income.^49^ Similarly, transitioning to organic farming or reduced-tillage methods often requires significant upfront investments in equipment, certification processes, and marketing know-how, which may be out of reach for smaller farms. In contrast, larger agricultural enterprises are better positioned to absorb these costs and benefit from economies of scale.^49^^,^^50^ Policymakers must therefore tailor environmental targets to address these disparities, ensuring that vulnerable agrifood systems are not left behind.

One significant challenge in creating equitable environmental policies lies in the disconnect between regional, national, and international agricultural policies and the realities faced by farmers at the local level. Policymakers often rely on broad frameworks to set environmental targets, such as those outlined in the EGD or the 2030 Agenda for Sustainable Development of the United Nations.^2^^,^^38^ While these frameworks are essential for global coordination, they may overlook localized challenges and opportunities, such as those faced by agricultural systems recognized as Globally Important Agricultural Heritage Systems (GIAHS) or Cultural Landscapes by UN Agencies.^51^^,^^52^ To safeguard the area’s cultural and historical value these sites must comply with specific landscape restrictions, often permitting only minimal interventions. More in general, the effectiveness of large-scale agri-environmental policies is often undermined by the so-called “spatial scale mismatch” between the organizational levels of agricultural management and those needed by local ecological processes.^53^ Local case studies and region-specific data are crucial for crafting policies that align with both environmental goals and the socio-economic needs of farmers. However, integrating such localized insights into broader frameworks remains a challenge due to the lack of efficient communication channels between policymakers and regional stakeholders. The disconnect between environmental policies and their implementation is often exacerbated by communication gaps among policymakers, scientists, and farmers. These gaps create inefficiencies and unintended consequences that hinder the success of sustainable agriculture initiatives.

### Divides between policy-makers, scientists, and stake-holders

Scientific research provides the foundation for evidence-based policy decisions. However, the complexity of scientific findings often fails to translate into actionable policies. Scientists typically present solutions designed for ideal conditions, while policymakers must navigate political, economic, and social constraints. This disconnect can potentially lead to policies that are either too generalized to address specific issues or overly ambitious, making them difficult to implement. For instance, precision agriculture technologies have demonstrated remarkable potential in improving resource use efficiency and reducing environmental impact. Despite their promise, these technologies remain underutilized due to a lack of alignment between scientific recommendations and policy incentives. In regions with prevailing smallholder farms, policies promoting these technologies often fail to address the infrastructural deficiencies and affordability issues that hinder their adoption.^54^ Bridging this gap require more collaborative efforts between scientists and policymakers to co-develop solutions that are both scientifically sound and pragmatically feasible. Regular dialogues, interdisciplinary task forces, and policy-relevant research agendas can ensure that scientific insights directly inform actionable policies. Even when policies are well-informed by science, their implementation often falls short due to a lack of engagement with farmers. This misalignment can create tensions between private and public interests, as farmers, despite being informed about climate change and environmental problems, tend to prioritize financial stability, which does not always align with broader environmental sustainability goals.^23^ Economic subsidies, a common tool for incentivizing sustainable practices, frequently fail to address the right problems or reach the farmers who need them most. Subsidies and incentives to increase production efficiency might prioritize large-scale systems, leaving small-scale farmers without access.^55^ In addition, farmers are more likely to adopt sustainable practices when they feel that policies allow a gradual transition, maintaining income stability and market access.^56^ To this end, policies should be inspired by farmer inputs and feedbacks, ensuring that subsidies and incentives are both relevant, economically accessible and easily complied. The gap between scientists and farmers further complicates efforts to promote sustainable agriculture. Research institutions and academia often produce innovative solutions to address agricultural challenges, but these solutions frequently fail to reach farmers or resonate with their practical needs. Conversely, farmers possess valuable experiential knowledge that can inform research but is rarely integrated into scientific agendas.^57^ Despite huge innovation steps have been taken, many of them are not easily transferred to stakeholders. For instance, a key challenge in remote sensing remains improving the transferability of methods between scales, minimizing inconsistencies across datasets and models.^25^ In addition, the cost-effectiveness of these technologies and data acquisition at the farm level is hardly accessible on a large scale.^16^ Similarly, many AI techniques are confined to simulation studies, underscoring the need for real-world applications. Additionally, AI’s contributions have mainly focused on monitoring, while limited applications have been implemented in harvesting and cultivation activities.^29^ Water conservation practices are often implemented at the experimental level, their adoption remains low in real-life scenarios, highlighting the need for clear communication of agronomic and environmental benefits to local farmers before application.^36^ In precision irrigation providing high-quality data, there is no cost-effective method to obtain high-quality soil, meteorological and evapotranspiration data and measure percolation losses.^15^ Scientific advancements in producing affordable and accessible tools for farmers would enable the widespread implementation of innovative and effective production systems.

## Framework bridging the gap between science, policy, stakeholders

**Figure 3:**
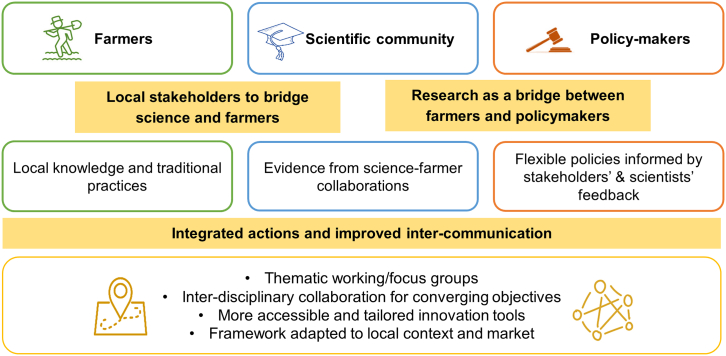
Conceptual flow diagram of the new proposed framework for agriculture under climate-change scenarios

The complex challenges of achieving environmental sustainability while preserving the socio-economic well-being of farmers require an integrated and flexible framework. This framework must address the communication gaps between policymakers, scientists, and farmers while recognizing local vulnerabilities, traditions, and socio-economic needs. By empowering local stakeholders, building trust through collaboration with scientific institutions, and ensuring policies are flexible and informed by ground realities, it is possible to apply solutions that satisfy farmers’ needs and align with global goals in terms of food security, namely SDG2 (Zero Hunger), and broader environmental targets, such as SDG13 (Climate Action) and SDG 15 (Life on land).^2^ In this section, we outline key strategies for bridging these divides and creating a cohesive, future-oriented framework (Figure 3), while also acknowledging the main remaining limitations and challenges.

### Integrating economic efficiency and agricultural policies

From an economical perspective, many of the environmental benefits provided by agriculture, such as carbon sequestration or biodiversity, are public goods and positive externalities often undervalued or overlooked by markets, resulting in market failures.^58^ To correct these, policy instruments, such as payments for ecosystem services, carbon pricing, and regulation frameworks aim to create economic incentives for sustainable practices.^59^^,^^60^ However, agricultural policies involve unavoidable trade-offs^61^: any change in land use, subsidies, or environmental regulations will likely produce unequal impacts on stakeholders. Moreover, policy frameworks should be designed with an understanding of how decisions are made under uncertainty, especially considering the current climate change scenario.^62^ Decision theory helps explain how farmers assess risk, respond to incentives, and may resist change when benefits are long-term or uncertain,^63^ considering the variety of preferences, capacities, and risk perceptions of stakeholders, anticipate behavioral responses to incentives, and support policy choices when outcomes are uncertain. In addition, even economically sound policies should be designed considering social acceptability.^64^ Thus, effective policy must acknowledge heterogeneity in risk perceptions, preferences, and capacities among stakeholders.^62^ Since farmers make decisions under uncertainty and are influenced by both short-term constraints and long-term risks,^65^^,^^66^ policy frameworks should not only provide incentives but also consider context-specific adaptation.

### Flexible policies tailored to local contexts

Effective environmental policies must be adaptable, allowing for regional variations in agricultural practices, climate conditions, and socio-economic realities. Blanket solutions often fail to account for the diversity within farming communities, leading to resistance or unintended negative consequences. In addition, societal priorities related to food security, climate action, and nature conservation differ across contexts, resulting in varying optimal approaches. Given the inevitable presence of conflicting interests between private (i.e., citizens and farmers) and public actors, it is essential to assess a range of policy options, including second-best alternatives.^67^ In particular, agricultural policies should increase farmers’ active contributions to large-scale environmental protection, enhancing their role as key agents of sustainable change. Flexible policies can address this by incorporating mechanisms for local customization and continuous feedback. Policies should begin with a comprehensive assessment of local vulnerabilities, such as frequent droughts, erosion, salinization, etc. To further guide interventions, flexible frameworks could incorporate localized data, such as soil fertility levels, crop health, and water availability. The adoption of participatory vulnerability assessments, where farmers themselves highlight challenges and priorities, can ensure that solutions are relevant and grounded in reality.^68^^,^^69^ Traditions and cultural practices often play a significant role in farming systems, influencing crop choices, resource management, community resilience and overall rural livelihood. Policymakers must respect and incorporate these traditions into environmental strategies. In fact, studies of agricultural performance following extreme climate events highlight that resilience is strongly associated with high on-farm biodiversity typical of traditional farming systems.^17^ Therefore, reviving traditional management systems of biodiversification, soil management, and water harvesting, can be both economically and environmentally beneficial. Additionally, flexible policies should consider the socio-economic needs of farming households, while also accounting for negative externalities.^70^ Subsidies, credit access, and the market should align with both environmental objectives and farmers’ income stability, ensuring that no group is disproportionately burdened during the transition to sustainable practices.

### The role of local stakeholders in bridging science and farmers

Local stakeholders, including water management authorities, farmer associations, and local officers, are pivotal in connecting science with farming practices. These actors can serve as intermediaries, translating complex scientific insights into actionable strategies, and building trust between farmers and researchers. Trust is a crucial step toward successful policy implementation. Local stakeholders, deeply embedded in their communities, are well-positioned to act as mediators, facilitating dialogue and ensuring transparency. For instance, water management authorities can organize workshops and involve farmers in the design, testing, and dissemination of scientific innovations.^71^ Similarly, being part of a farmers’ organization enhances farmers’ access to shared knowledge, enabling mutual learning and the exchange of effective strategies to address climate change.^72^ Local stakeholders can also play a critical role in co-creating economically viable solutions that balance farmers’ needs with environmental goals. To ensure competitiveness, stakeholders must also take market dynamics into account. In this sense, farmers’ organizations, associations, and cooperatives can facilitate the access to market providing business and management knowledge, physical infrastructure, and operational support, particularly in the case of small-scale producers.^73^ Local governments and organizations can collaborate in supporting supply chains for traditional products or sustainable cultivation techniques, providing incentives for private investments in processing and marketing opportunities.^74^ This approach not only promotes economic resilience but also ensures that environmental policies do not abruptly reduce farmers’ profitability.

### Research as a bridge between farmers and policymakers

Scientific research is essential for informing evidence-based policies, but researchers must be fully aware of the socio-economic of agriculture and farming communities. Collaborative research projects, where farmers actively participate in the design and implementation of studies, can generate insights that are both scientifically rigorous and practically applicable. Incorporating farmers as “co-researchers” ensures that their experiential knowledge informs scientific inquiries^19^ related to key topics, such-as breeding, water management, pest control, soil management, etc. These programs not only produce tangible outcomes but also strengthen trust between farmers and the scientific community. Research outputs must be translated into policy-relevant insights. This requires clear communication channels between researchers and policymakers, with a focus on synthesizing findings into actionable recommendations. Evidence from science-farmer collaborations, such as techniques to optimize soil and water use, can directly inform regulatory frameworks.^57^ In order to ensure the feasibility of translating research outputs into effective policies, it is crucial to organize workshops, facilitate communication and exchanges of information, and guarantee access to key scientific knowledge for farmers and stakeholders. These activities involve transaction costs that must be properly accounted for, and the associated time and resources should always be included in project proposals as essential components of research activities.

### Integrated actions and improved inter-communication

A unified approach to addressing environmental and socio-economic challenges demands integrated actions and enhanced communication across all stakeholder groups. This involves collaboration between disciplines, open dialogue, and creating platforms for continuous engagement. Establishing multi-stakeholder platforms can facilitate collaboration and knowledge-sharing among farmers, scientists, policymakers, and private sector actors. These platforms can be structured as regional councils, thematic working/focus groups, addressing specific issues, such as soil health or water management, depending on the main local constraints. Regular meetings, ideally supported by digital tools, can ensure that insights are exchanged and policies remain adaptive. In this sense, digital tools, such as mobile apps and online portals, can play a significant role in bridging communication gaps.^75^ These platforms can disseminate localized weather forecasts, market trends, and best practices to farmers while collecting data on their experiences and feedback. Spreading such technologies, especially in underserved regions, can enhance the reach and impact of sustainable farming initiatives. Digital literacy and training are critical for empowering all stakeholders to engage effectively^76^: farmers and farmer associations should have access to proper communication and training in digital tools applied to sustainable practices.

### Limitations and challenges in the applicability of the proposed framework

While the proposed framework is intended as a set of general guidelines and best practices, its implementation is not without challenges. Below, we outline several key limitations that must be addressed to ensure the framework’s adaptability to specific local contexts.

• Barriers in farmer education and access to technologies: small and medium-sized farms face significant challenges in adopting digital and climate-smart technologies. These barriers include underdeveloped markets, restricted access to capital, and limited knowledge or education, which hinder their ability to engage with innovative solutions effectively. Addressing these disparities is crucial to ensuring equitable access to technological advancements.
• Limited access to knowledge in remote and vulnerable areas: both farmers and the scientific community face challenges in accessing and sharing localized, practical knowledge essential for addressing the specific needs of remote and vulnerable areas. In these contexts, farmers often face challenges in accessing actionable guidance tailored to their unique circumstances, while researchers may overlook these areas due to their perceived lower priority or encounter difficulties in collecting and disseminating relevant data. This gap in knowledge exchange undermines the implementation of adaptive practices.
• Scientific limitations and uncertainties: applying idealized scientific results to real-world scenarios can present significant gaps. In particular, discrepancies may arise when models and algorithms are used in specific locations with insufficient, inaccurate, or poorly integrated input data.^77^ As a result, the proper contextualization of scientific findings can become challenging.
• Framework adaptability to diverse contexts: our proposed framework is primarily informed by a European perspective, where decades of CAP have shaped agricultural practices and agrifood systems. However, it is important to acknowledge the considerable variability in the application of EU policies even within European countries, and the fact that beyond Europe differing agricultural priorities and resource constraints prevail, particularly in low and middle-income countries and/or conflict zones. As such, this framework is intended as a flexible guideline, requiring contextual adaptation to effectively address specific regional needs and challenges, which may place varying emphasis on food security, climate protection, and nature conservation.
• Divergent priorities within the scientific community: the scientific community itself exhibits divergent opinions on prioritizing actions to contrast climate change in agriculture. While some research emphasizes environmental conservation, others focus on advancing sustainable agricultural intensification, leading to conflicting approaches depending on the scientific background.^14^ Greater collaboration and dialogue within the scientific community are essential to establish common ground, emphasizing actionable strategies that leverage farmers as stewards of the landscape rather than merely resource exploiters.

## Conclusions and way forward

In conclusion, to bridge the divides between policymakers, scientists, and farmers, a future framework must prioritize flexibility, inclusivity, and integration. Tailoring policies to local contexts, empowering local stakeholders, and fostering collaboration through participatory research and multi-stakeholder platforms, are important steps toward aligning environmental goals with farmers’ socio-economic realities. Improved communication, supported by education and digital tools, can support the development of solutions that are actionable, equitable, and sustainable. Scientists play a crucial intermediary role in engaging and educating farmers, while also highlighting how supporting their environmental efforts can enhance the effectiveness of climate protection measures. This integrated approach can contribute to achieving a resilient agricultural sector that balances the need to feed a growing population and safeguard the planet for future generations. Future steps to adapt the proposed framework to different cultural and socio-economic contexts should focus on overcoming technological and economical barriers, improving scientific knowledge of regional and local environmental issues, and fostering greater collaboration within the scientific community to align objectives aimed at achieving environmental, social, and economic sustainability in agriculture. In particular, where resources are limited or unevenly distributed, priority should be given to traditional practices that also deliver valuable ecosystem services (e.g., soil management techniques and water harvesting methods). New technologies should be integrated as well, but only after a careful evaluation of their actual benefits and adequately considering the potential trade-offs.^78^ In this regard, further research is needed to address scientific uncertainties, especially concerning the application of AI and idealized models to real-world contexts.

## Acknowledgments

The work is supported by the Italian Agritech National Research Center and received funding from the European Union Next-GenerationEU (PIANO NAZIONALE DI RIPRESA E RESILIENZA (PNRR)– MISSIONE. 4 COMPONENTE 2, INVESTIMENTO 1.4– D.D. 1032 17/06/2022, CN00000022). This work was also supported by PHITO (Platform for Helping small and medium farmers to Incorporate digital Technology for equal Opportunities) Horizon EU project n. 101084332. This manuscript reflects only the authors’ views and opinions; neither the European Union nor the European Commission can be considered responsible for them.

## Author contributions

Conceptualization, P.T. and A.G.; writing—original draft, A.G.; writing—review & editing, P.T; supervision, P.T.

## Declaration of interests

P.T. is a member of the iScience Advisory Board.
